# Characteristics of Potential Protein Biomarkers Extracted with 10% TCA from Blood Serum of Non-Hodgkin’s Lymphoma and Multiple Myeloma Patients

**DOI:** 10.22088/BUMS.6.4.235

**Published:** 2017-11-11

**Authors:** Severyn Myronovskij, Olga Shalay, Veronika Spivak, Rostyslav Stoika, Yuriy Kit

**Affiliations:** 1 *Department of Regulation of Cell Proliferation and Apoptosis, Institute of Cell Biology, National Academy of Sciences of Ukraine, Lviv, Ukraine* *.*; 2 *Laboratory of Immunocytology and Genetics of * *Blood Tumors , Institute of Blood Pathology and Transfusion Medicine, National Academy of Medical Sciences of Ukraine, Lviv, Ukraine.*; 3 *Biological Faculty of Ivan Franko Lviv National University, Lviv, Ukraine.*

**Sir,**

Blood serum has been extensively explored as a source of biomarkers (1, 2). For concentration of minor protein(s) and depletion of abundant blood serum proteins a 2,2,2-trichloroacetic acid (TCA) precipitation procedure is frequently applied (3). However, a significant amount of proteins may be present in the TCA extracts, and these proteins are often not studied. Recently, we have shown that a TCA- extracted fraction obtained from blood serum of the multiple sclerosis patients contains two proteins that were identified by the MALDI TOF/TOF as blood serum albumin (BSA) and a short form of the unconventional myosin lc (sMyo1C) (4). We also demonstrated that the TCA-extracted fractions isolated from blood serum of the multiple sclerosis patients contain IgGs or their heavy chains (5). These IgG molecules have not been detected in the TCA-extracted fractions isolated from blood serum of healthy human donors and patients with the systemic lupus erythematosus or the rheumatoid arthritis (5). Here we compare the proteins isolated from blood serum of the non-Hodgkin’s lymphoma and multiple myeloma patients, extracted through earlier described procedure including precipitation by10% TCA followed by precipitation with acetone (4,5). To this aim, peripheral blood serum samples of 12 patients (35–55-year old; 7 men and 5 women) diagnosed with multiple myeloma and 12 patients (*35–55*-year old; 6 men and 6 women) diagnosed with non-Hodgking’s lymphoma were studied. All patients were newly diagnosed and did not undergo chemotherapy. Informed consent was obtained from all patients, and the study was approved by the Review Board of the Bio-Ethics Committee at the Institute of Blood Pathology and Transfusion Medicine, NAMS of Ukraine, in accordance with the regulations of the Ministry of Health Protection of Ukraine. 12 blood serum samples from healthy donors (25–35-year old; 3 men and 9 women) were provided by the Lviv Regional Blood Service Center and were used as controls.

**Fig. 1.**
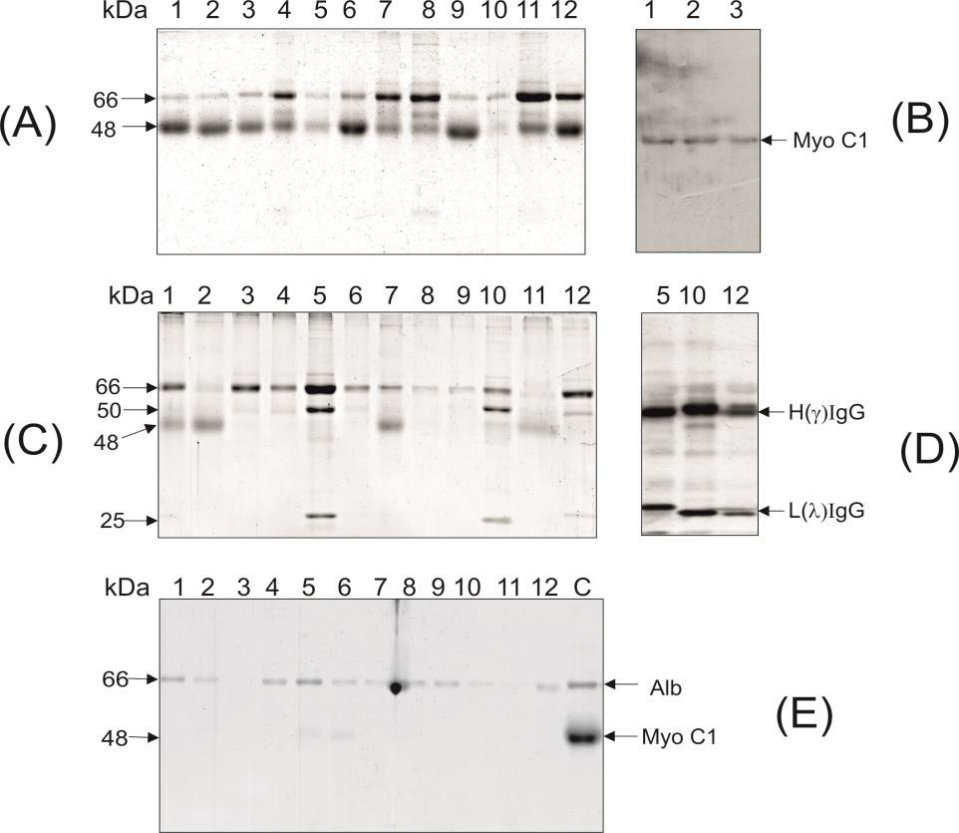
Characteristics of the proteins extracted with 10% TCA from the blood serums of non-Hodgkin’s lymphoma, multiple melanoma patients and healthy donors. A: SDS-PAGE electrophoresis of the TCA-extracted proteins from blood serum of the lymphoma patients; B: Western-blot analysis of TCA-extracted blood serum proteins using anti-48 kDa Myo 1c mice antibodies; C: SDS-PAGE electrophoresis of the TCA-extracted proteins from blood serum of the multiple myeloma patients; D: Western-blot analysis of the TCA-extracted proteins using anti-human IgG (whole molecule) rabbit antibodies; E: SDS-PAGE electrophoresis of TCA –extracted proteins from blood serum of the healthy donors (lane C: 48 kDa Myo C1 protein, purified from blood serum of multiple sclerosis patients (ref.4) was used as a marker

According to our protocol (5), 1 ml of blood serum was diluted 2-fold with the phosphate buffered saline, and then 100% TCA was added to 10% final concentration. After 30 min incubation at-20°C, the solution was subjected to centrifug-ation for 15 min at 10,000 g. The supernatant fraction containing TCA-soluble compounds was isolated and mixed with the acetone in 1:6 ratio followed by incubation for 18 h at-20C.

The results of the SDS-PAGE electrophoresis demonstrated that the TCA-extracted fraction obtained from blood serum of the non-Hodgkin’s lymphoma patients mainly contained proteins with 66 and 48 kDa molecular mass (Figure1A). Recently, proteins with similar molecular mass have been identified in the TCA- extracted fraction of blood serum of the multiple sclerosis patients as the BSA (66 kDa), and a 48 kDa fragment of the unconventional myosin 1C (sMyo1C), respectively (5). To confirm that the 48 kDa protein isolated from blood serum of the non-Hodgkin’s lymphoma patients was the sMyo1C, the Western-blot analysis based on using the affinity purified anti- human sMyo1C mouse antibodies was applied (7). The results of binding of specific antibodies with the 48 kDa band proved the identity of sMyo1C (Figure. 1B).

In contrast to the TCA-extracted fraction of blood serum of the non-Hodgkin’s lymphoma, where the BSA and sMyo1C were the prevalent proteins, in 3 out of 12 patients diagnosed with multiple myeloma, additional polypeptides with 50 and 25 kDa molecular mass were found (Figure 1C). We suggested that these may be the heavy and light chains of the IgG molecule. The Western-blot analysis based on the use of monospecific anti-human IgG rabbit HRP-conjugated antibodies (7) proved that suggestion (Figure 1D). The electrophoretic analysis of 12 healthy donors is shown in Figure 1E. It revealed the presence of low amount of sMyo1C within two samples of the TCA- extracted proteins (lanes 5, 6), whereas the heavy and light chain polypeptides of IgG were not detected in all samples.

It should be noted that the TCA-soluble form of the IgGs have been earlier detected by us as a rare component of the TCA-extracted polypeptides isolated from blood serum of the multiple sclerosis patients, and it was not detected in healthy human donors or patients with the systemic lupus erythematosus and the rheumatoid arthritis (5). Multiple myeloma and non-Hodgkin’s lymphoma are the lymphoproliferative diseases able to secrete various proteins having diagnostic and prognostic value (8-11). Elevated level of myosin IC isoform b (48/myo1c) was also detected in blood serum of patients with rheumatoid arthritis, and Alzheimer's disease (12). Low level of this protein was detected in blood serum of healthy humans but not in blood serum of patients with type 1 diabetes, cirrhosis, thyroiditis, and recurrent miscarriage (12). The level of sMyo1C in blood serum correlates with certain type of human diseases that may have diagnostic value. The myeloma proteins have obvious interest since they contain abnormal immunoglobulins or immunoglobulin light chains that are produced in excess by an abnormal monoclonal proliferation typical to multiple myeloma plasma cells (10). Why some IgGs possess the ability to dissolve in TCA, while others precipitate in the same conditions, remains unclear. The high positive charge or hyperglycosylation could serve as an explanation to the properties of these IgG molecules (5).

In conclusion, blood serum of the autoimmune and hemato-oncological patients contains specific TCA-soluble polypeptides (sMyo 1C - 48 kDa fragment of the unconventional myosin1C or/and heavy and light chains (50 and 25 kDa) of the IgG molecule. We suggest that these polypeptides could serve as the potential molecular biomarkers, helpful for estimation of progressing and severity of those diseases. Further investigations are needed to shed light on these issues.
